# A Tangible Multimodal Sensing Interface for Collaborative Task Engagement in Children With Autism Spectrum Disorder: Cross-Sectional Mixed Methods Feasibility Study

**DOI:** 10.2196/86264

**Published:** 2026-10-15

**Authors:** Won Kim, Chaeyeon Lim, Minwoo Seong, SeungJun Kim

**Affiliations:** 1 Department of AI, Gwangju Institute of Science and Technology, Dasan Building 201, Cheomdangwagi-ro 123, Gwangju, 61005, Republic of Korea, 82 62 715 5352; 2 Department of AI·SW (Computer Engineering), Chosun University, Gwangju, Republic of Korea

**Keywords:** children with autism spectrum disorder, interaction and sensing technologies, engagement, collaborative and social serious game, machine learning

## Abstract

**Background:**

Social engagement is critical for the developmental progress of children with autism spectrum disorder (ASD). While tangible technologies and serious games (SGs) show promise, a dual-purpose platform that both facilitates collaborative interaction and enables sensing of collaborative task engagement is required. Existing tangible interfaces often lack integrated sensing capabilities, while devices designed for complex input are typically not optimized for the sensory and interaction needs of this population.

**Objective:**

This study aims to present InteractiSense, a tangible prototype designed to support collaborative play while enabling engagement estimation for children with ASD through embedded multimodal sensing. Our primary objectives were twofold: (1) to evaluate the prototype’s preference, usability, and workload, and (2) to assess the feasibility of its embedded sensor data in generating valid inputs for preliminary machine learning (ML)-based engagement classification.

**Methods:**

We developed InteractiSense, a spherical, soft-textured prototype embedded with 7 physio-behavioral sensors, alongside a collaborative SG promoting key social skills, and evaluated it against a familiar Xbox controller as a practical baseline. Fifteen children with ASD (mean age 11, SD 3 years; 10 males and 5 females) participated in a cross-sectional feasibility evaluation using a within-subjects mixed methods design. Preference data were analyzed using the McNemar test and Bayesian analysis, and usability and workload scores were compared using 2-tailed paired *t* tests. Qualitative caregiver interviews on InteractiSense and the SG were analyzed using thematic analysis, with device-related themes contextualizing quantitative findings during interpretation. To validate sensing feasibility, we analyzed the signal-to-noise ratio (SNR) of the sensor data and trained a preliminary extreme gradient boosting (XGBoost) model, comparing it with baseline models to classify binary engagement states using video-coded ground truths.

**Results:**

InteractiSense showed a tendency toward higher preference and comparable usability and workload metrics relative to the Xbox controller. Caregivers associated its round shape, soft texture, and simplified configuration with favorable perceptions and noted peer collaboration during the SG and interest in home use. Signal quality varied across sensing modalities: selected modalities provided analyzable data, whereas audio quality was low and pressure data were excluded from SNR and ML analyses. XGBoost achieved 83% (CI 0.76-0.90) balanced accuracy in binary engagement classification, exceeding random and majority-class baselines.

**Conclusions:**

These findings provide initial evidence that InteractiSense can serve as a dual-purpose platform supporting collaborative play and engagement-aware sensing within the same tangible interface. Unlike prior systems focused on either interaction support or separate monitoring, InteractiSense combines embedded multimodal sensing with a social play interface in one platform for children with ASD. This study offers a proof-of-concept for extending tangible play systems toward engagement-related sensing and inference, with implications for future therapeutic and educational tools that monitor play and support adaptive intervention. However, feasibility was shown only at a preliminary level, requiring validation in larger cohorts and over longer observation periods.

## Introduction

### Background

Social engagement, defined as coordinated attention toward both a social partner and a shared physical activity [1], plays an important role in supporting developmental progress in children with autism spectrum disorder (ASD), serving as a foundation for developing communication and interpersonal skills [2]. Due to delays in cognitive, motor, and social development, they are prone to boredom and disengagement, which can hinder their participation in daily and social activities [2,3]. Therefore, measuring and understanding engagement is essential for improving and managing engagement [4]. Within the peer-based gameplay context examined in this study, we focus on a narrower, context-specific construct, collaborative task engagement, defined as observable participation in a shared game task with a peer; this construct does not necessarily require direct peer-oriented attention or interaction at every moment. Accordingly, engagement-promoting technologies for children with ASD have been explored through interactive and tangible approaches [5], such as soft interactive social prototypes [6,7], wearable companions [8], and Mazi, a soft, round tangible prototype developed to encourage spontaneous and collaborative play [9]. These can be beneficial in fostering social skills by increasing engagement through spontaneous interaction [10] initiated by children. Studies have focused on leveraging tangibility with soft, textile-based approaches that are nonvulnerable, easy to handle, and particularly appealing to children with ASD, who often show a preference for physically graspable and soft materials that provide comforting tactile sensations, helping sustain active engagement [7,11,12]; yet most of these efforts have centered on enhancing engagement, with limited attention to how participation in collaborative tasks can be systematically assessed.

Measuring engagement and sustained participation can inform the assessment of social-skill development in educational or therapeutic settings for children with ASD [4]. Prior work has integrated sensors into interactive, tangible, and commercial systems, including SuperpowerGlass [13] and sensorized toys [14], and explored soft interactive companions with the potential to support social skills [8]. Sensor-integrated forms such as spheres, tori, and soft dolls with feedback have also been proposed for nonautistic children [15] and may be particularly relevant to ASD settings requiring engagement monitoring and, potentially, tailored automated support [16]. Because engagement is complex, multimodal sensing is important; however, commercial devices often constrain sensor integration, conflict with engagement-monitoring requirements, and lack adaptability to ASD-related needs such as sensory sensitivity and safe, familiar, and robust form factors [17-19]. These challenges motivate a tangible platform that combines multimodal sensing and interaction while accounting for user reception, usability, and workload. Accordingly, we developed InteractiSense as a preliminary dual-purpose prototype and evaluated it through expert interviews and a user study with caregiver postinterviews. The prototype was developed to estimate collaborative task engagement during a peer-based collaborative serious game (SG), referring specifically to participation in the shared game task rather than broader social engagement.

The goal of this study is to evaluate the feasibility of InteractiSense as a dual-purpose platform across 2 dimensions. First, interaction feasibility requires the prototype to yield usability and workload metrics comparable to, or exceeding, those of an Xbox controller, a baseline device reflecting users’ long-term exposure. Second, sensing feasibility requires the embedded multimodal sensors to capture data with sufficient signal fidelity to support a preliminary machine learning (ML) model for engagement classification. To achieve this, we address the following two research questions: (1) How do children with ASD perceive and interact with InteractiSense, a tangible interface for social and collaborative gameplay, compared with an Xbox controller, in terms of preference, usability, and workload? (2) How feasible can InteractiSense be in generating sensor signals that serve as valid inputs for implementing ML models to estimate collaborative task engagement in children with ASD?

### Related Works

#### Measuring Engagement

Engagement is a multidimensional construct encompassing mental, behavioral, and emotional components [20]. Sensor-based assessment is promising for children with ASD, who may have difficulty self-evaluating and expressing their experiences [21,22], while multimodal sensing can improve engagement prediction [4,23]. Accordingly, InteractiSense integrates diverse physio-behavioral and peripheral modalities to examine their individual use for engagement assessment.

The sensor metrics used are as follows: galvanic skin response (GSR), which measures skin conductance, detects affective and attentional states associated with engagement [24,25]. Heightened GSR serves as an indicator of increased engagement during interactions, making it a promising metric for identifying the engagement status [26]. Heart rate (HR), measured through changes in beats per minute, showed potential for detecting engagement in nonautistic students during structured writing activities [27]. Skin temperature (ST), a thermal response indicator, can also be used to assess arousal and attentional states [28,29]. Inertial measurements, comprising accelerometers and gyroscopes, quantify physical involvement and serve as an indicator of engagement by capturing behavioral patterns, activity levels, and synchronization with others [30-32]. In addition, physical involvement, such as grasping and progressing actions, is associated with engagement, as tasks and given interactions like applying pressure [12,33,34] and providing user input through buttons connect fundamental sensory and motor activity [35,36]. Therefore, pressure and button inputs were included for their high relevance in assessing engagement. Audio amplitude generated by children with ASD correlates with their engagement and attention during social activities [37,38], offering a noninvasive method for monitoring behavioral states as a measure of perceived loudness [28]. In this study, we integrated these multidimensional sensors into InteractiSense, which we propose to enable engagement estimation while supporting interactions for children with ASD.

#### Necessity of Novel Interacting and Sensing Prototype

Video games were among the earliest digital interventions used to entertain and encourage participation among children with ASD. Their associated console controllers have since become familiar in daily life and have been linked to behavioral regulation and reductions in stress and stereotyped behaviors [39,40]. However, familiar devices such as the Xbox controller may activate leisure-oriented associations [41], potentially constraining openness to SGs for educational, therapeutic, skill-building, or structured collaborative purposes [42,43]. Their familiar form may therefore constrain interpretive flexibility, motivating alternative interaction modalities [44,45]. Such alternatives should provide usability comparable to or better than that of console controllers [46] without imposing greater task-related demands [47], making the Xbox controller an appropriate reference interface [48].

Recent systems have combined interaction support with social engagement monitoring for children with ASD. Ward et al [32] used accelerometers to examine interpersonal synchrony in theatrical interactions, while Lanini et al [14] used a commercial wireless sensorized toy to assess ASD-related behaviors. Using a commercial toy rather than a wearable or camera-based system also offers a less invasive approach intended to improve generalizability and promote spontaneous engagement [9]. Varma et al [49] used smartphone-based gaze measures to identify social engagement during semistructured parent-child gameplay. However, these systems often rely on single modalities [14,32], limiting their ability to capture the multifaceted nature of engagement. Although multimodal sensing can improve engagement prediction [20], it remains underexplored in tangible prototypes [49]. The transitional wearable companions of Ozcan et al [8] similarly emphasize soft, interactive, and tangible designs intended to support social-skill development [11,12], child-initiated interaction, and multimodal feedback. Although tangible and spontaneous interactions can benefit peer engagement [9], systems combining them with multimodal engagement sensing remain scarce [12]. This gap motivates dual-purpose prototypes that support both interaction and sensing in SG contexts, providing a basis for future research on targeted interventions.

## Methods

### Research Design Overview

This study presents the development and expert-informed refinement of InteractiSense, a tangible prototype integrating interaction and multimodal sensing, together with a collaborative SG designed to support peer-based collaborative task engagement. Its feasibility was evaluated in children with ASD using a cross-sectional, within-subjects mixed methods design, in which each child completed both the InteractiSense and Xbox controller conditions with condition order alternated across pairs. Interaction feasibility was assessed through device preference, usability, and workload measures supplemented by postsession qualitative interviews, whereas sensing feasibility was assessed through sensor-signal quality analysis and preliminary ML-based engagement classification.

### Ethical Considerations

This human participants study involving children with ASD was reviewed and approved by the Institutional Review Board (IRB) of the Gwangju Institute of Science and Technology (GIST; approval number HR-61-04-04, including subsequent approved amendments). The study was not exempt from ethics review, and no waiver of informed consent was used. All procedures were conducted in accordance with the IRB-approved protocol and applicable institutional ethical guidelines. The approved protocol covered the involvement of professionals from multiple disciplines and the participation of children with ASD and their legal guardians.

Prior to participation, the experimental procedures and the role of the proxy user in the usability and workload evaluations were explained to each child’s parent or legal guardian. Written informed consent for each child’s participation was obtained from their parent or legal guardian. In addition, parents or caregivers who participated as proxy users provided written informed consent for their voluntary participation and the use of their data for research purposes. Parents or legal guardians were informed that participation could be discontinued at any time at the request of the child, legal guardian, or therapist. The experiment commenced only after the child’s willingness to participate had been confirmed.

Each participant received monetary compensation equivalent to approximately US $35, which was transferred to a designated bank account. All collected data were de-identified immediately upon collection, and each participant was assigned a unique identification code. Personally identifiable information was stored separately from the research data in a secure, password-protected database, with access restricted to the primary researcher. No individual participant or user is identifiable in any image included in the manuscript or supplementary materials.

### Inclusion and Exclusion

Children were eligible to participate if they were aged between 6 and 15 years and had either a diagnosis of ASD or ASD-related characteristics identified by the special education center. Children younger than 6 years or older than 15 years were excluded to maintain a sample within the predefined developmental age range. During the initial screening, 2 children younger than 6 years and one high-school–aged student older than 15 years were excluded based on these criteria.

### Participant Characteristics

Following screening, 15 eligible children were recruited from a local special education center in accordance with the IRB-approved recruitment procedures (Table 1). Participants ranged in age from 6 to 15 (mean 11, SD 3) years and included 10 males and 5 females. They represented a broad range of functional profiles, including 7 children with co-occurring intellectual disability (ID) and 2 with co-occurring attention-deficit/hyperactivity disorder (ADHD).

**Table 1.** Demographic and clinical characteristics of the study participants^a^.

Participants	Chronological age (years)	Functional age (years)	Sex	Disability	Disability severity^b^	School grade^c^	Hand length (cm)
P^d^1	10	7	Male	ASD ^e^+ ID^f^	L^g^-3	G^h^-4	14.9
P2	15	10	Male	ASD	L-5	G-9	17.2
P3	10	10	Female	ASD	L-5	G-4	14.7
P4	9	9	Female	ASD + ID	L-3	G-2	14
P5	12	8	Female	ASD + ID	L-4	G-5	15.5
P6	14	8	Female	ASD + ID	L-3	G-7	16.3
P7	14	14	Male	ASD + ADHD	L-2	G-8	16.6
P8	8	8	Male	ASD + ADHD	L-1	G-1	13.3
P9	6	4	Male	ASD	L-5	Kinder^i^	12.1
P10	8	5	Male	ASD	L-3	G-1	13
P11	14	3	Female	ASD + ID	L-2	G-7	16.4
P12	15	7	Male	ASD	L-5	G-8	17
P13	14	5	Male	ASD	L-4	G-7	16.2
P14	13	8	Male	ASD + ID	L-3	G-8	15.9
P15	11	11	Male	ASD + ID	L-4	G-6	15.1

a Standardized clinical severity levels, consistent with *Diagnostic and Statistical Manual of Mental Disorders, 5th edition* (*DSM-5*) criteria, could not be uniformly documented due to jurisdictional variations in regional disability registration policies. In lieu of official classifications, caregiver-reported severity levels were included to provide an overview of the participants’ functional and behavioral status.

b Symptom severity was quantified through a self-report measure, where respondents indicated the degree of impairment associated with the child’s specific disability type (survey question: “Please indicate the severity level of the symptoms pertaining to the child’s disability category identified in the previous section.”)

c To ensure a continuous measure of educational development, we adopted a standardized grading system ranging from G1 to G9. This corresponds to the cumulative years of schooling in South Korea, encompassing both primary (Grades 1‐6) and secondary (Grades 1‐3) levels.

d P: participant.

e ASD: autism spectrum disorder.

f ID: intellectual disability.

g L: level.

h G: grade.

i Kinder: kindergartener.

### InteractiSense Design

#### InteractiSense Prototype

The InteractiSense adopts a spherical form, as its round and soft design minimizes the risk of injury for children with ASD during play [15]. This design also aligns with the natural communicative and collaborative mechanisms of socialization [50], providing a familiar and safe structure that fosters spontaneous interactions in both their daily life and therapeutic settings [9,51]. Our design process considered prior concepts from the TangToys workshop, soft animal-shaped dolls, a torus, a cube, and a sphere, which aimed to support communication and well-being [15]. The torus was excluded due to constraints in sensor deployment, and the cube due to concerns about edge sharpness [51]. A spherical structure was selected not only for its physical safety but also due to its capacity to accommodate various forms of interaction (eg, holding, pressing, and rotating) [9,51].

Designed with an outer diameter of 18 cm to support 2-handed manipulation for a typical 10-year-old (whose average hand length is approximately 15 cm), the body was fabricated using 3D printing to ensure precise placement and integration of 7 sensor positions (Figure 1). The 18 cm measurement refers to the overall spherical enclosure rather than the required grip span. To enhance its affordance and tactile experience, the handle of InteractiSense features finger grooves for a secure and comfortable grip, while its surface is covered with yellow-colored [51,52] and felt fabric, a soft textile and comforting color that reflects the characteristics of transitional objects (Figure 1) [53,54]. Furthermore, a hook-and-loop hand strap is attached to ensure a stable mount, promoting self-directed interaction by the child. The prototype (Multimedia Appendix 1) was also presented in an extended abstract for a public demonstration [55]. In this study, InteractiSense is evaluated more broadly through a mixed methods feasibility approach, including preliminary ML-based engagement classification.

**Figure 1.**
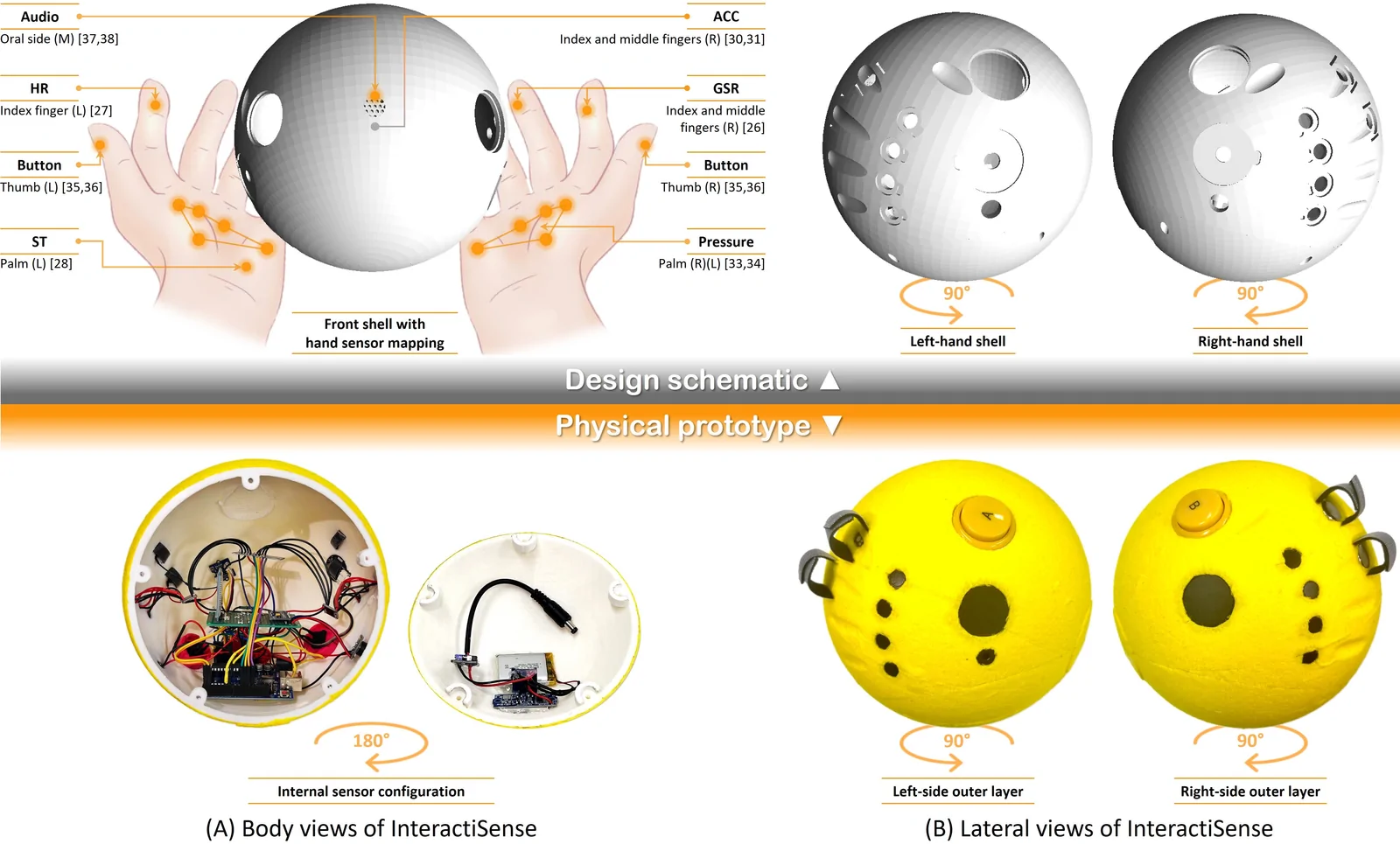
InteractiSense (A) on-hand sensor placement and (B) lateral views. The label (L) indicates sensor locations corresponding to the left hand, and the label (R) indicates those corresponding to the right hand. ACC: accelerometers; GSR: galvanic skin response; HR: heart rate; ST: skin temperature.

#### Technical Specification

InteractiSense integrates 7 physio-behavioral sensors aimed at monitoring engagement status (Figure 1 and Multimedia Appendix 2). Sensor data are wirelessly transmitted via low-energy Bluetooth at 2.1 Mbps with a 10-meter range (≤314 m^2^ circular range), ensuring real-time multimodal transmission without data loss in spacious environments. The power uses a 1200 mAh lithium polymer battery, accounting for total power consumption and supporting continuous operation (≤10 hours), allowing for use in home settings and extended interaction periods.

### Collaborative and Social SG Design

The selection of game elements in “Treasure Hunters” was based on existing literature regarding evidence-based behavioral interventions for children with ASD. Clinical studies indicate that foundational social skills serve as essential precursors to social communication development [56,57]. Furthermore, research on interactive systems demonstrates that incorporating these clinical targets into collaborative gameplay mechanics effectively supports shared focus and physical peer engagement [58,59].

The SG was designed to enhance the social skills through gaming modules incorporating turn-taking, joint attention, and imitation [60]. These skills were integrated into a collaborative gameplay framework called “Treasure Hunters,” developed using Unity3D, where players progress along a rail track by completing gaming modules (Figure 2). Turn-taking involves taking actions alternately with another player, such as performing an action and then waiting for the other player’s turn [59,61], while joint attention emphasizes focusing on a shared goal [62,63]. Both are integrated into the timing slider game, where players defeat monsters with button (B) and mine treasures with button (A) (Figure 3), either by taking turns or working together (Figure 2A). Imitation involves following guidance, embracing each other before reaching the fall zone on the rail (Figure 2C) [61,63]. During gameplay, pairs of children played through 21 gaming modules, with each module assigned using a balanced Latin square. Between gaming modules, we incorporated a Game Refresher, a monotonous running-on-rails scene, intended to serve as a sensory break and provide cognitive rest [64]. The game ends by reporting the performance from the previous gameplay. The game featured 3 difficulty levels, with stronger-looking monsters, higher-value treasures, and reduced scores at higher levels. Considering the hypersensitivity and sensory delays of ASD [65], we prioritized simplified interactions and streamlined feedback.

**Figure 2.**
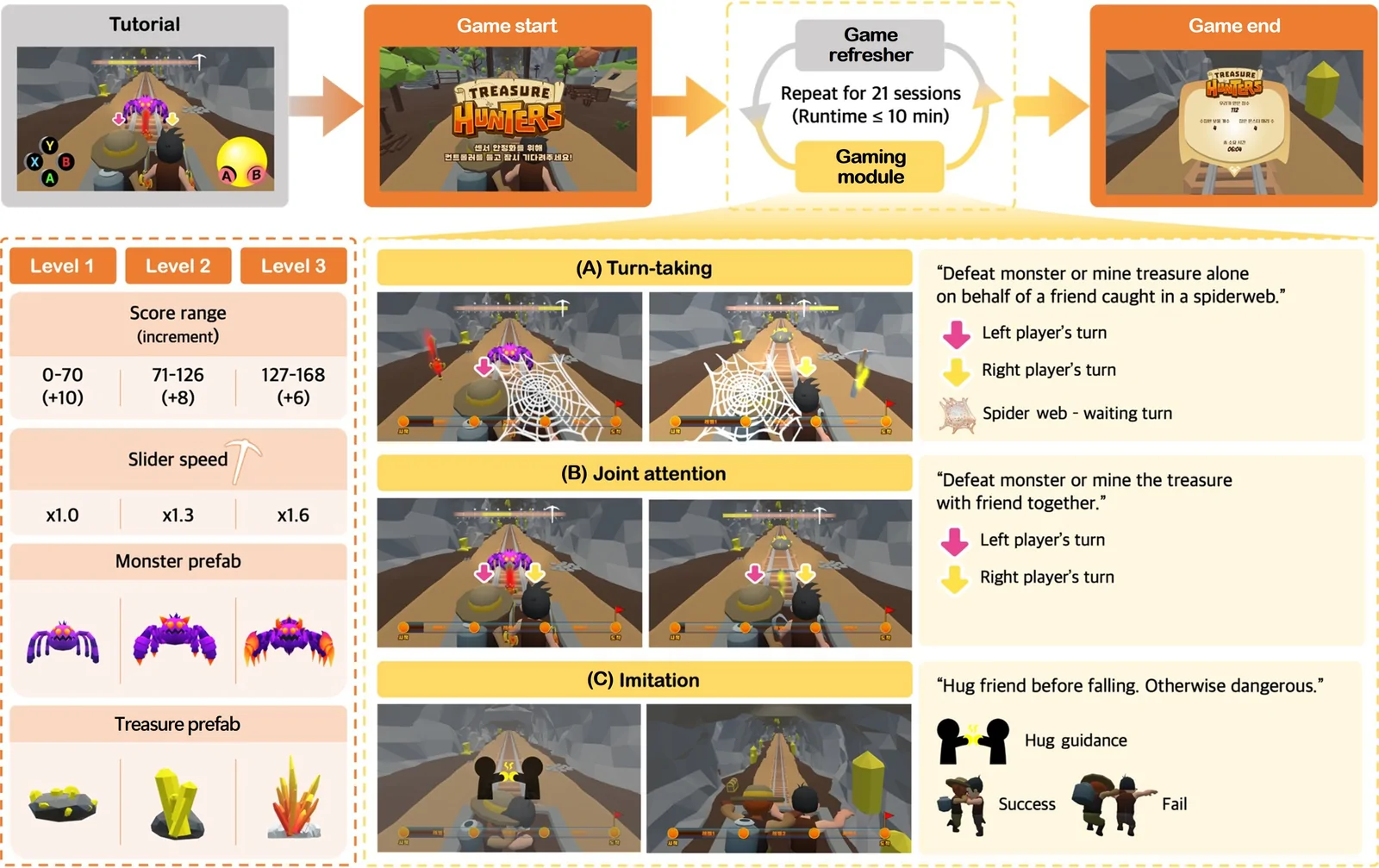
Serious game procedures and description with difficulty level design.

**Figure 3.**
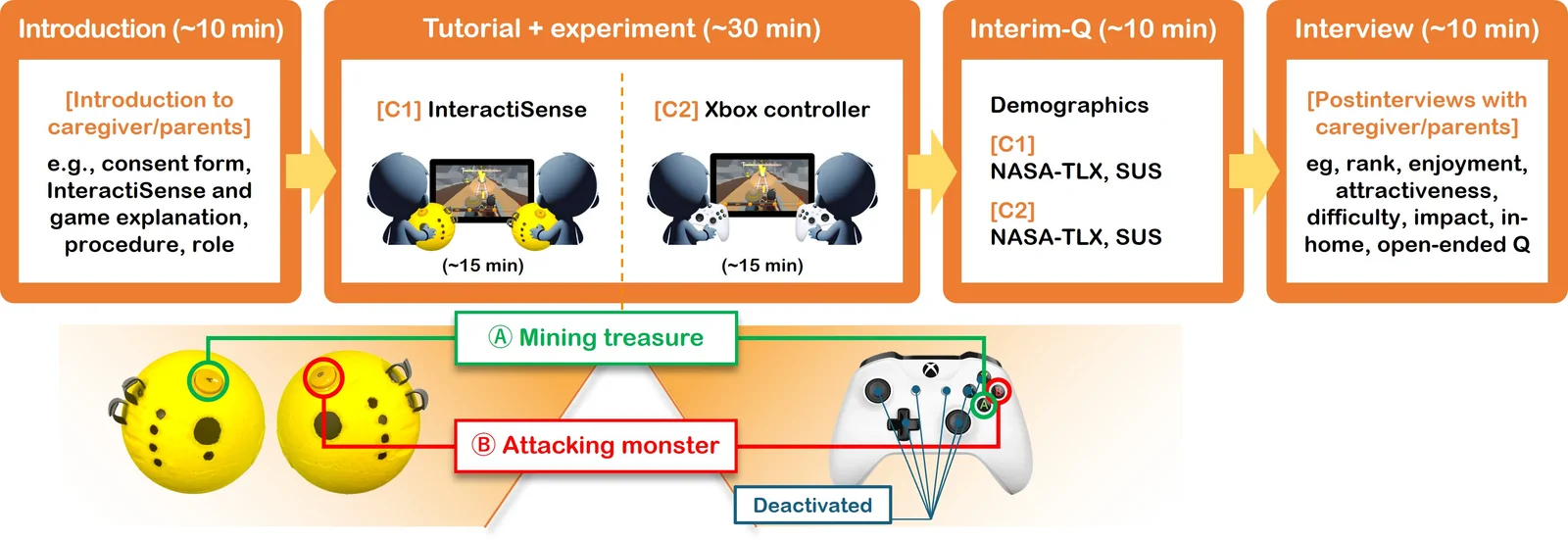
Experimental procedure and controller mapping. (Top) The overall sequence of the experiment. (Bottom) Mapping configurations for the serious game task in each condition. For C2 (Xbox controller), only the A and B buttons, the most accessible for right-hand operation, were activated, while all other buttons were disabled to ensure consistency.

### Expert-Involved Iterative Design

#### Expert Interview

We conducted expert interviews and a pilot test for iterative design with 3 teachers who work in special school (>17-year experience). The experts were given an overview of the study and participated in a pilot test of InteractiSense and the SG, assuming a child with moderate to severe ASD. We then requested a quantitative evaluation of InteractiSense through questionnaires on workload NASA Task Load Index (NASA-TLX) [47] and System Usability Scale (SUS) [46], as well as qualitative feedback on the prototype and SG, including insights on how they were perceived and the specific contexts in which the prototype could be applied in real classroom settings.

#### Expert Insights and Findings

The need for enhancing the tutorial was identified by 3 experts to better support tutorial and explicit visual guidance, fostering gameplay confidence in children with ASD. We incorporated visual feedback through button-pressing animations that map the player’s input interaction to each device (Figure 2 [Tutorial]). In the initial version of the game, a small spiderweb was displayed to the player waiting for the other’s turn. This was later improved by enlarging the spiderweb and adding a colored arrow to indicate the player’s turn. The importance of emphasizing success feedback was raised, leading to enhancements, confetti effects, success reflections on in-game characters (embracing effects), and reinforced cheering sounds. Incidentally, experts noted that soft, ball-like objects are often used in therapeutic settings to promote gentle, tactile engagement, which informed our decision to adopt a spherical design. Quantitative evaluation revealed that experts’ workload and usability scores were positioned as moderate-to-high workload (mean 57, SD 17) and excellent usability (mean 84, SD 11.8), with both showing appropriate difficulty and ease of application.

### Data Collection

#### Data Collection Setting

Following an iterative expert review, the study involving children with ASD was conducted at the Dream Tree Children Education Center in Gwangju Metropolitan City, Republic of Korea, from January 16 to 22, 2025. The center is a community-based institution that provides educational and support services for children with special educational needs, including programs addressing basic motor function, physical fitness, and physical and psychological health. Participants with ASD were recruited from children attending the center. Recruitment began approximately 1 week before the experimental sessions. During this period, study information was posted at the center and provided to parents and caregivers of potentially eligible children. Parents and caregivers who were interested in the study were invited to indicate their willingness for their child to participate. Potential participants subsequently underwent preliminary screening for eligibility, and the final study sample was selected according to the predefined inclusion and exclusion criteria.

The experimental setup is illustrated in Figure 4. Paired children were seated side by side in front of a 55-inch display connected to an Apple MacBook Pro with an M4 Pro chip running the Unity3D-based SG. InteractiSense and the Xbox controller were connected to the MacBook Pro via Bluetooth for gameplay input, with InteractiSense additionally transmitting its embedded sensor data to the computer. Each session was recorded from front, side, and rear viewpoints using 3 cameras, providing complementary views for subsequent behavioral coding and review.

**Figure 4.**
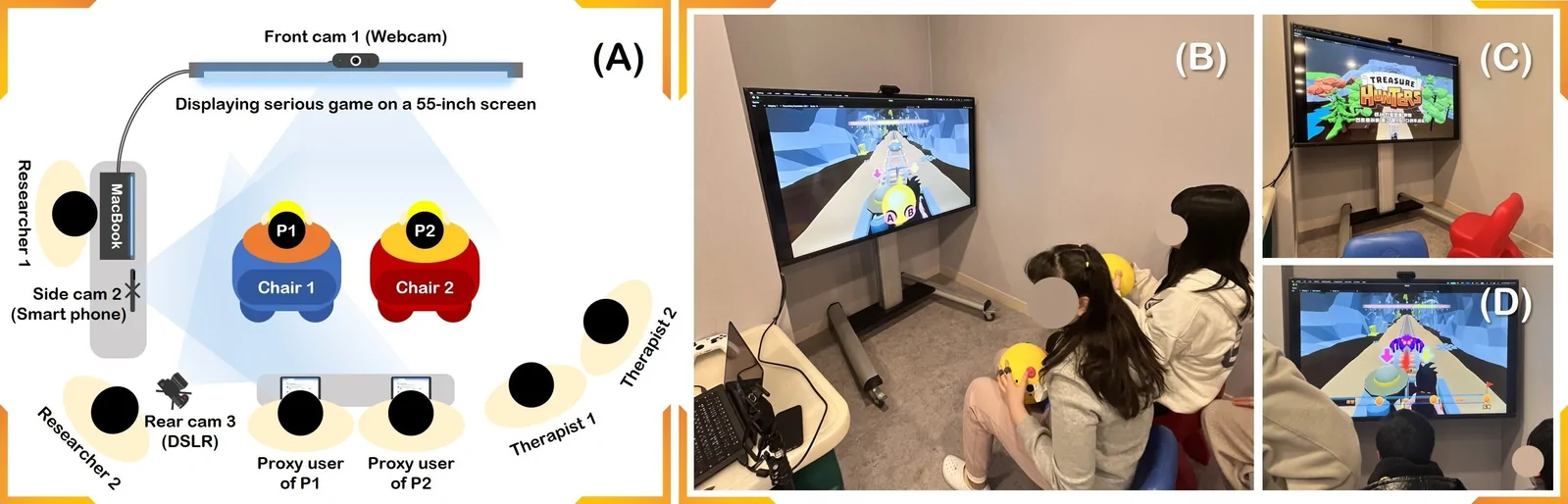
Experimental setting for the InteractiSense evaluation. (A) Schematic floor plan detailing the spatial arrangement of the screen, device, participants (children with autism spectrum disorder, therapists, proxy users, and experimenters) and video-recording cameras (eg, a webcam, an iPhone 12 mini smartphone, and a digital single-lens reflex camera). (B) Side view of the tutorial session, illustrating participants familiarizing themselves with the InteractiSense device and the serious game. (C) Unoccupied physical setting of the main screen and the children's seating area. (D) Observation view from the perspective of proxy users and therapists monitoring the children during a collaborative task. DSLR: digital single-lens reflex camera.

#### Sampling Procedures

Accompanying parents participated as proxy respondents to provide assessments on behalf of children with ASD, or, when parents were unavailable, caregivers attended in their place [21,22]. They were informed about the experiment and completed consent and demographic forms, while a tutorial was conducted to familiarize participants with the experiment (Figure 3). Paired children were assigned 2 conditions (C1: InteractiSense, C2: Xbox controller) that alternated the order for each pair, following a within-subjects design. Given its widespread familiarity and established role in gaming, the Xbox controller was selected as the baseline input device, as its form [66] and usage are already well-integrated into the experiences of children with ASD [48], a decision further supported by expert feedback indicating its common use in regionally implemented educational and recreational purposes. For the Xbox controller (C2), the interaction was specifically mapped to the A and B buttons, the most accessible for right-hand operation, while all other buttons were deactivated to ensure a consistent control assessment (Figure 3). Using this recognized standard allowed us to evaluate InteractiSense as a promising option for practical applications. After each condition, proxy users completed interim questionnaires. During the experiment, 2 therapists and proxy users observed the children and managed unexpected actions. After completing all assigned experimental conditions, the accompanying parent, or a caregiver when the parent was unavailable, participated in an approximately 10-minute face-to-face semistructured interview. The postsession semistructured interviews were conducted by one of the researchers, who had no prior relationship with the study participants. Children with ASD who were able and willing to respond were also invited to share their views, which were incorporated into the qualitative findings. With consent, the interviews were audio-recorded, transcribed, and analyzed thematically to identify recurring themes. During the analysis, plausible alternative interpretations were considered, and no substantively new themes emerged from the later interviews, indicating that thematic saturation had been reached. The complete interview guide is provided in Multimedia Appendix 3.

#### Sample Size, Power, and Precision

This cross-sectional study focused on evaluating the interaction and sensing feasibility of InteractiSense. The planned sample size was 25 children, and the final sample comprised 15 eligible children. Recruitment was restricted to a single special education center to maintain observational consistency. Data from 8 participants were retained for the preliminary ML analysis. Uncertainty in the interaction-related estimates was summarized using 95% CIs for the preference odds ratio (OR) and device-specific mean usability and workload scores, together with a 95% credible interval for the Bayesian estimate of preference probability.

#### Measures and Covariates

The measures evaluated in this study addressed 2 dimensions of feasibility: interaction feasibility and sensing feasibility. Device condition (InteractiSense vs Xbox controller) was the within-subjects experimental factor for the interaction-feasibility comparisons. Interaction feasibility was assessed through device preference, usability using SUS [46], and workload using NASA-TLX [47,67,68]. Postsession semistructured interviews further assessed perceived enjoyment, task performance and assistance needs, device-related facilitators and barriers, participation in the shared task, potential home use, and suggested improvements.

Sensing feasibility was assessed through signal-to-noise ratio (SNR) analysis of the analyzable sensor modalities and preliminary ML-based engagement classification. Sensor-derived features from the retained modalities served as predictors, while video-coded binary engagement states (engaged vs not engaged) served as the classification target. Balanced accuracy was the primary classification metric, supplemented by precision, recall, *F*_1_-score, and confusion matrices. Participant characteristics were reported descriptively in Table 1. No covariate adjustment was applied to the device-condition comparisons. To reduce potential order effects, game-module order was counterbalanced using a balanced Latin square, and device-condition order was alternated across pairs.

### Data Sources

Data were collected from children with ASD and their accompanying parents or caregivers during the study. Demographic and clinical characteristics were obtained from questionnaires completed by parents or caregivers. Device preference, usability, and workload were assessed using study questionnaires, with SUS and NASA-TLX completed by proxy users after each condition. Qualitative data were obtained from postsession semistructured interviews with parents or caregivers, with additional comments from children who were able and willing to respond. Interviews were audio-recorded and transcribed; the interview guide is provided in Multimedia Appendix 3.

Sensor data were obtained from the modalities embedded in InteractiSense, with sampling details provided in Multimedia Appendix 2. Engagement labels were derived from video recordings and annotated in EUDICO Linguistic Annotator (ELAN) by 2 independent coders (WK and CL) according to the criteria. Only labels agreed upon by both coders were retained for analysis.

### Engagement Classification

We assessed the validity of the embedded sensor signals and their potential to support engagement-level classification, using a preliminary ML implementation aimed at evaluating feasibility rather than optimizing performance. Given the small sample size and class imbalance, we did not assume that a more complex model was inherently warranted; instead, we used baseline comparisons to examine whether the sensor-derived features contained predictive structure beyond simple decision rules.

#### Annotation and Ground Truths

Researcher-coded engagement labels for children with ASD were derived from video recordings. Each session was recorded using 3 cameras, and the ELAN software was used to annotate engagement status. The start and end times of each behavioral event were marked with second-level granularity. Each event was assigned to 1 of 2 labels (0: not engaged, 1: engaged) based on the specific criteria (Table 2). Two independent coders (WK and CL) performed the annotations, and interrater agreement was evaluated following [69], yielding an agreement score of 0.94. Only events where both coders assigned the same label were retained as the ground truth for subsequent analyses. These annotations served as the ground truths for training and evaluating the classification models.

**Table 2.** Operational definitions and behavioral criteria for engagement labeling within a peer-based social gaming context, detailing specific indicators for “Not engaged” (0) and “engaged” (1) states across visual, verbal, and physical dimensions.

Engagement	Conceptual definition	Operational criteria
0 (Not engaged)	A state where the participant is detached from the social gaming context or the shared task with a peer.	Any of the following behaviors occurred: (1) gaze: looking at areas unrelated to the game content, (2) verbalization: speaking about topics unrelated to the game (excluding mumbles or babbling), (3) physical: releasing the InteractiSense device, (4) external: intervention by an experimenter, parent, or teacher.
1 (Engaged)	A state of sustained participation in the social gaming context and collaborative task.	All other instances where the participant is actively interacting with the game and none of the “Not engaged” criteria are met.

#### Data Segmentation and Feature Construction

Given the limited number of available datasets, ML methods were used, particularly those suited for imbalanced distributions and small-sample contexts (Figure 5). Input data were segmented into 25-sample windows with a step size of 0.5 seconds. To mitigate the scarcity of minority-class windows that would result from a strict majority rule, each window was labeled as 0 if the proportion of label-0 frames was greater than or equal to 0.2, and as 1 otherwise. This threshold corresponds to at least 5 label-0 frames (=0.17 seconds within a 25-sample window) and was empirically chosen to preserve the minority class while maintaining temporal consistency with observed engagement dynamics.

**Figure 5.**
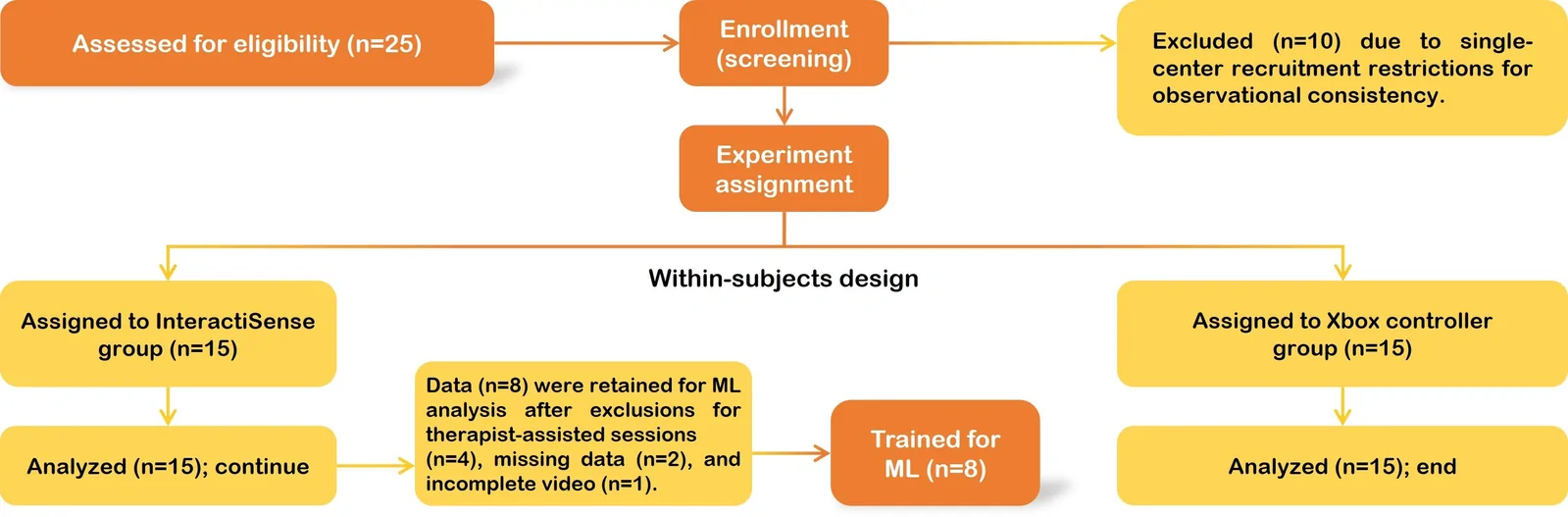
Participant flow diagram. The flowchart details the enrollment process, the within-subjects allocation to both experimental conditions, and the subsequent data filtering steps for the machine learning analysis. ML: machine learning.

Feature vectors concatenated the raw samples with 12 summary statistics (mean, SD, minimum, maximum, range, median, IQR, slope, energy, root-mean-square, zero-crossing rate, and dominant frequency ratio) computed for each sensing channel. The sensing modalities used as ML inputs included inertial measurements (orientation: Yaw, Pitch, Roll; ACC: ACC_X, ACC_Y, ACC_Z; gyroscope (Gyro): Gyro_X, Gyro_Y, Gyro_Z), physiological signals (HR, GSR, and ST), and audio amplitude data. Pressure input was excluded because its sensing configuration required a low activation threshold to register brief and localized contacts, which led to a high proportion of missing values and rendered the data unsuitable for regression-based analysis and window-level labeling. Data were collected from 15 children with ASD, and samples from 8 participants were retained for ML analysis due to therapist-assisted sessions (n=4), missing data from connectivity issues or gameplay interruptions initiated at the child’s request (n=2), and annotation infeasibility due to incomplete video coverage of the child’s participation (n=1). The final dataset comprised 5745 windows after segmentation and labeling, with an imbalanced distribution (class-0: 139, 2.4%, class-1: 5606, 97.6%, ≈1:40 ratio). Model training was conducted on 4596 windows (class-0: 111, class-1: 4485).

#### Classification Model and Evaluation

Extreme gradient boosting (XGBoost) was selected for its capacity to handle structured sequential inputs, generate probabilistic outputs for threshold tuning, and incorporate class-imbalance handling [70]. Class imbalance was handled at two levels: (1) class-prior correction using *scale_pos_weight* = *N*_0_/*N*_1_, where *N*_0_ and *N*_1_ are the counts of labels 0 and 1 in the training split, and (2) sample-level weights to give higher weight to minority-class and borderline windows near the labeling threshold. For comparison, 3 baselines were also implemented: majority, which predicts the most frequent class in the training split; and random (uniform), which samples labels with equal probability; and logistic regression, a linear classifier included as a stronger baseline. Performance was assessed using balanced accuracy (primary), precision, recall, *F*_1_-score, and confusion matrices to characterize class-wise behavior.

Model evaluation used Group 8-fold cross-validation to prevent data from the same participant appearing in both training and validation folds. Hyperparameters were tuned via random search over standard XGBoost ranges for tree depth, minimum child weight, subsample ratio, column subsample ratio, 𝛾, and L_1_/L_2_ penalties (Multimedia Appendix 4). Early stopping monitored validation log-loss within each fold. The decision threshold τ was selected to maximize balanced accuracy using the validation data only and was then fixed before application to the test set; no test-set information was used for threshold selection. Predictions from 3 models trained with different random seeds were averaged. All stochastic processes, including data splitting, parameter search, and model training, were initialized with fixed random seeds to ensure reproducibility.

### Analysis

#### Data-Analytic Strategies

Quantitative user preferences were analyzed using the McNemar test and ORs for discordant pairs because the preference responses were paired within participants across the 2 device conditions, thereby accounting for the within-participant dependency of the observations. The preference analysis was supplemented by a Bayesian analysis using a uniform Beta(1,1) prior, representing no a priori preference for either device. Proxy user-reported usability and workload scores, measured using the SUS and NASA-TLX, respectively, were compared using 2-tailed paired *t* tests, with the proxy user as the unit of analysis (n=15). To validate physiological data fidelity, the SNR was calculated via an autocorrelation function with a second-order polynomial fit.

Qualitative interview data on InteractiSense and the SG were analyzed using thematic analysis. Quantitative findings and interview themes were integrated during interpretation in the Discussion, where caregivers’ accounts of device characteristics and familiarization were used to contextualize preference and usability findings.

#### Methodological Integrity

During the qualitative analysis, plausible alternative interpretations were considered. No substantively new themes emerged from the later interviews, which was interpreted as evidence of thematic saturation within the dataset. Findings concerning InteractiSense and the SG were grounded in interview accounts and illustrated with quotations from caregivers and children who contributed comments.

## Results

### Quantitative Evaluation

Out of 15 participants, 10 (67%) indicated a preference for InteractiSense and 5 (33%) for the Controller (Table 3). McNemar test showed that this difference was not statistically significant, *P*=.30. The OR for discordant pairs was 2 (95% CI 0.62-7.46). Updating a uniform Beta(1,1) prior with the observed preference data yielded a Beta(11,6) posterior distribution. The posterior mean was 0.65 (SD 0.11), with a 95% credible interval of (0.41-0.85) and a posterior probability of 0.89 that the preference probability exceeded 0.50.

**Table 3.** Comparison of device preference, usability, and workload between InteractiSense and the Xbox controller (n=15).

Measures	InteractiSense	Controller	Test statistics	*P* value
Preference, n (%)	10 (67)	5 (33)	McNemar test^a^	.30
Posterior preference probability^b^				
Mean (SD)	0.65 (0.11)	—^c^	—	
95 % credible interval	0.41‐0.85	—	—	
P (preference >0.50)	0.89	—	—	
SUS^d^ score			–0.22^e^ (14)	.83
Mean (SD)	65.8 (14.7)	67.1 (16.4)	—	
95% CI	56.91‐74.63	57.20‐77.04	—	
NASA-TLX^f^ score			–0.37 (14)	.71
Mean (SD)	46.6 (9.1)	47.9 (9.7)	—	
95% CI	41.04‐52.08	42.06‐53.84	—	

a 2-sided McNemar test; odds ratio for discordant pairs 2 (95% CI 0.62-7.46).

b Beta(11,6) posterior distribution of the probability of preferring InteractiSense, derived from a uniform Beta(1,1) prior. CrI: credible interval.

c N/A: not applicable.

d SUS: System Usability Scale. Scores for both devices fell within the marginal range of acceptability.

e 2-tailed paired *t* test.

f NASA-TLX: NASA Task Load Index. Scores for both devices corresponded to a somewhat high workload level.

Regarding performance, both measures showed only subtle differences, with the controller yielding higher usability and InteractiSense leading to lower workload. However, 2-tailed paired *t* tests did not indicate significant differences: for usability, InteractiSense (mean 65.8, SD 14.7) and Controller (mean 67.1, SD 16.4) yielded (*t*_14_=–0.22, *P*=.83); for workload, InteractiSense (mean 46.6, SD 9.13) and Controller (mean 47.9, SD 9.7) yielded (*t*_14_=–0.37, *P*=.71). Separate analyses of the NASA-TLX subscales and SUS items also yielded no significant condition differences. The results indicate that InteractiSense demonstrated usability and workload levels that were slightly lower but similar to those of the robust baseline (ie, the familiar Xbox controller). Its potential as a tool for children with ASD was identified, supported by additional insights from postexperiment interviews.

### Qualitative Evaluation

Our postinterviews gathered insights into children’s preferences, feedback, and the applicability of InteractiSense and the SG. Caregivers noted that while children were not accustomed to InteractiSense at first, they recognized its distinct advantages over the Xbox controller. One caregiver (P8) remarked:

The child seemed to prefer the simple button configuration of InteractiSense, as it was easier to map the game elements to the buttons. With only two buttons, once the child becomes accustomed to it, using InteractiSense will be easier than a traditional controller (also echoed by P1, P11 and P12).

Children’s preference for InteractiSense was not solely due to its functionality; the device’s physical attributes, such as its round shape, soft touch, and bright color, also played a noticeable role. The round shape, in particular, was noted as offering psychological comfort and aiding fine motor development, such as movements of the fingers and palms (P2, P3, and P7). One child shared his excitement, exclaiming, “I liked the egg yolk!” However, some caregivers pointed out limitations due to the fixed size of InteractiSense and suggested that a more flexible and soft design could enhance its usability (P8, P10, and P11). Given the Xbox controller’s familiarity to most children (P4), caregivers noted that once adapted, the prototype’s simplified form and operation could yield practical usability benefits (P6 and P9). While its usability scores were comparable to those of the controller, the streamlined design of our prototype, featuring tangible elements such as its round shape, bright appearance, and soft texture, was perceived more favorably for children with ASD compared to the controller.

The social and collaborative SG, integrated with InteractiSense, was noted for several advantages, one of which was its ability to encourage paired interaction, as caregivers appreciated its application for children with ASD, who often engage in solitary play due to their characteristics. Caregivers shared notable observations during gameplay, stating:

Children waited for their turns (P10), enjoyed collaborating (P1), and even encouraged distracted peers to rejoin the game (P12). One child, in particular, showed a positive change in behavior, remaining calm even when the game did not proceed as planned, which differed from their usual pattern (P6).

Expanding on these insights, several caregivers added:

This game seems to provide a medium for our children to learn collaboration, an advanced form of social interaction (P1), and foster closer relationships (P4, P5).

Following the game, P4 and P5, who were not initially close, were even observed holding hands, playing together, and sharing their belongings. More than 13 caregivers expressed their willingness to use InteractiSense, along with its functional games or educational content, if it were made available for home use, and highlighted its potential for use with other family members.

### Validation of InteractiSense Data

SNR was calculated in dB to validate data using raw signals, based on the autocorrelation function with a second-order polynomial fit. The average SNR values showed that ST yielded the highest (26.8 dB), followed by GSR (15.6 dB) and HR (15.7 dB), while audio exhibited the lowest (3.1 dB; Table 4). ST maintained continuous acquisition despite intermittent separation, which likely arises from its noncontact nature. In contrast, GSR and HR are contact-based modalities, driven by electrodes and optics, and are sensitive to detachment, looseness, and motion, which introduced artifacts and reduced SNR in segments. Audio yielded signals heavily mixed with ambient noise, as persistent background sounds from caregivers, therapists, and other children masked participant speech and reduced the effective SNR. The pressure modality produced sparse, discontinuous signals due to recurrent separation, and thus SNR analysis was not performed. These results indicate that ST provided consistently high-quality signals (>20 dB) [4], while HR and GSR yielded signals of sufficient quality for analysis, which aligns with thresholds considered acceptable for clinical practice (>14 dB) [71]. Potential improvements include optimizing sensor placement, considering alternative sensing mechanisms, and applying postsignal processing techniques (eg, frequency filtering and wavelet transform).

**Table 4.** Statistics on the signal-to-noise ratio of physiological signals collected from InteractiSense. All signal-to-noise ratio values are reported in dB. Quantiles (Q) represent the distribution of signal-to-noise ratio values.

Sensor metric (unit)	Mean (SD)	Min	Q15	Q25	Q50	Q70	Q95	Max
GSR^a^ (dB)	15.57 (7.28)	6.19	7.53	9.62	16.95	20.96	23.27	23.86
HR^b^ (dB)	15.74 (1.82)	13.5	14.22	14.59	15.46	16.83	18.12	18.45
ST^c^ (dB)	26.77 (6.79)	20.64	21.83	22.38	24.18	29.51	36.63	38.55
Audio amplitude (dB)	3.11 (1.36)	1.69	1.86	1.94	2.67	4.25	5.02	5.17

a GSR: galvanic skin response.

b HR: heart rate.

c ST: skin temperature.

### InteractiSense Classification

On the binary engagement classification task (engaged vs disengaged) for children with ASD, the XGBoost model achieved a balanced accuracy of 83% (Figure 6A). This value is higher than the random guess and majority baselines (both 50%), showing that the model surpasses trivial classifiers. This performance indicates that InteractiSense can serve as a feasible approach for distinguishing engagement states. In addition, class-wise recall reached 85.7% for the disengaged class and 80.2% for the engaged class, demonstrating discriminative performance for both classes despite the class imbalance (Figure 6B). Taken together, these results provide preliminary evidence of InteractiSense as a feasible prototype for differentiating engagement states in children with ASD.

**Figure 6.**
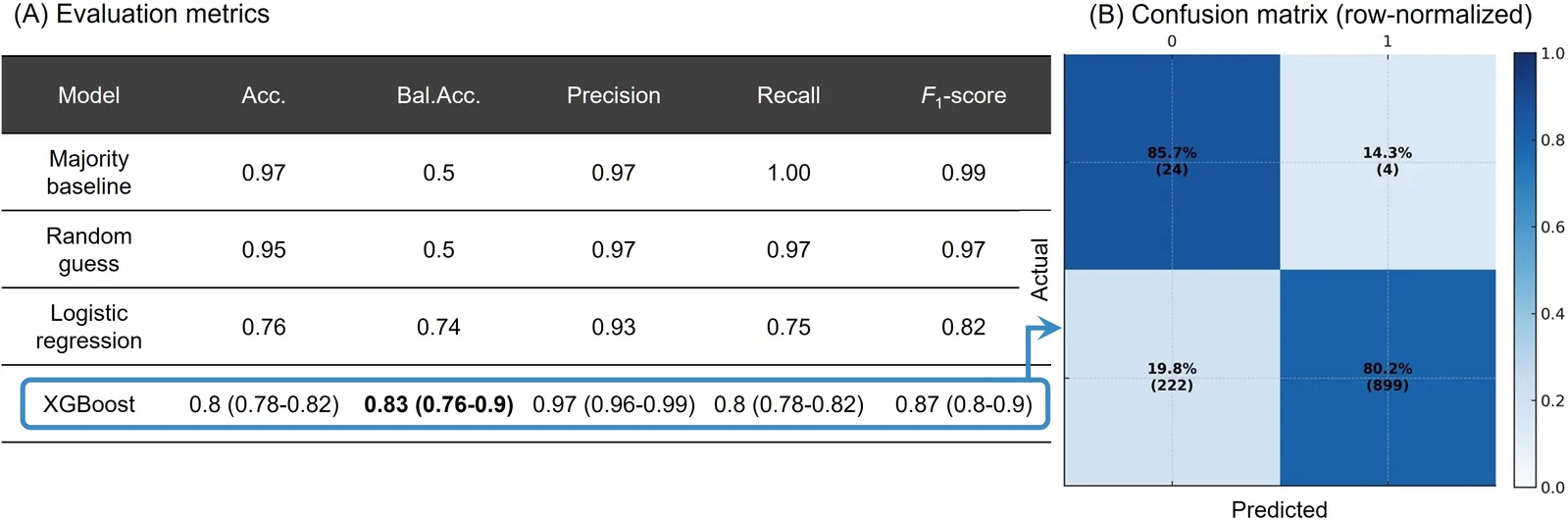
Model evaluation results. (A) Classification metrics of extreme gradient boosting and baseline methods. (B) Row-normalized confusion matrix. In (A), the column header Acc. denotes accuracy, and Bal. Acc. denotes balanced accuracy. XGBoost: extreme gradient boosting.

## Discussion

### Principal Findings

#### InteractiSense Reached Functional Parity With a Familiar Baseline While Eliciting Favorable Reception

In this short-term feasibility study, preference was included as an exploratory indicator of initial receptivity to the unfamiliar interaction concept and form factor, rather than as a proxy for usability or therapeutic effectiveness. InteractiSense tended to be preferred over the conventional Xbox controller, which served as the comparison baseline in this study, although remarkable differences were not confirmed; Bayesian analysis suggested a high probability that InteractiSense was favored, indicating favorable initial reception despite its unfamiliarity. In terms of usability, InteractiSense performed slightly below the conventional Xbox controller, whereas on workload it performed marginally above; however, neither measure indicated statistically significant differences. These outcomes suggest that InteractiSense achieved a level of performance comparable to the baseline. Considering that the Xbox controller represents a familiar and widely accepted interface for children with ASD [48], achieving functional parity in these short-term usability and workload measures supports the interpretation that the prototype is usable and holds potential for practical deployment in ASD-oriented play contexts.

The tendency for InteractiSense to be preferred over the Xbox controller was echoed in postinterview accounts. Caregivers emphasized that the simplified 2-button configuration reduced the effort required to map game actions, and that the prototype’s tangible qualities, its round shape, soft texture, and bright appearance, contributed to comfort and engagement, consistent with prior work showing that tangible designs with enriched sensory cues can promote attention and collaborative play among children with ASD [51]. These characteristics helped explain why children were reported to show enthusiasm for our device despite its unfamiliarity and their tendency to favor familiarity [36]. Some caregivers also noted that once children adapted, the streamlined design could offer practical advantages, although improvements in size and flexibility would be desirable. Together, the quantitative pattern and interview accounts converge to suggest that while InteractiSense achieves functional parity with familiar interfaces, its positive reception is more accurately attributed to its ability to become an object of interest for the children, a finding that aligns with prior work underscoring the centrality of user interest [72].

Given that the Xbox controller represented a robust baseline grounded in longer-term familiarity [36,48], whereas InteractiSense was evaluated after short-term exposure, the comparable pattern observed here may be interpreted as encouraging. However, this interpretation should be considered in light of the limited sample and the absence of repeated exposure over time, highlighting the need for further validation in a larger cohort.

#### InteractiSense Demonstrated Feasibility as a Sensing Platform for Engagement Classification

The integration of SNR analysis with the preliminary ML implementation indicated that selected InteractiSense modalities exhibited sufficient stability for engagement modeling, while signal quality varied across sensors. Among the sensing modalities, ST yielded consistently robust signals, even during movement, while HR and GSR provided valid and analyzable data, although somewhat less stable due to their susceptibility to detachment and motion artifacts [73,74]. The reduced stability observed for GSR is consistent with prior ambulatory GSR research showing that movement-related artifacts can compromise signal quality [75]. In the present setting, this contrast suggests a relative advantage of noncontact sensing in dynamic play contexts, while also highlighting the need for careful handling of contact-based modalities. Overall, these results indicate that InteractiSense can function not only as an interaction tool but also as a preliminary sensing platform that provides multimodal inputs for engagement modeling.

Building on this stability, preliminary ML indicated that InteractiSense holds potential to support binary classification of engagement states, with XGBoost performance exceeding baselines (eg, random guess, majority baseline, and logistic regression). Classification outcomes further indicated balanced accuracy above 0.8 across both classes, with disengagement classified with higher accuracy. However, given the inclusion of only 8 participants, the temporal dependence among windows, and the extreme class imbalance, the aggregate balanced accuracy should be interpreted cautiously and only as preliminary feasibility evidence. The model may also have captured movement- or sensor contact-related patterns associated with the annotated labels rather than engagement itself.

Valid sensing and preliminary classification together indicate that InteractiSense extends beyond its role as an interaction device, demonstrating a dual role as both a participatory social interaction medium and a sensing platform for engagement inference. In doing so, this work realizes the conceptual goals for such “TangToys” [15], while extending the capabilities of earlier sensorized toys [14] from single-modal behavioral observation to complex, multimodal engagement inference. The findings underscore its potential to guide monitoring approaches that may evolve into adaptive support strategies that, in line with the growing demand for interventions [4,76], can adjust the assistance provided based on a child’s inferred state of engagement [20]. This serves as a proof-of-concept for embedding engagement-aware sensing into tangible play environments, providing methodological direction for subsequent system development.

#### Perceptions of the Social and Collaborative SG

The integrated social and collaborative SG revealed further advantages beyond the device itself. Caregivers observed that the SG encouraged paired interaction among children who often prefer solitary play [77], with instances of turn-taking, peer encouragement, and calmer responses to setbacks. Such observations suggest that the game context provided a medium for practicing collaboration and building peer relationships, which are often difficult to nurture in children with ASD [59,78]. This finding aligns with a body of research suggesting that the clear rules and shared goals inherent in digital SGs can provide a crucial scaffold for children with ASD, simplifying the complex social demands of unstructured play [59,79]. Caregivers also expressed interest in adopting this type of content for home use, highlighting its potential to extend engagement beyond structured settings [80]. In sum, these accounts indicate that collaborative SGs integrated with novel interaction tools can amplify social affordances and point toward future directions for designing social play that fosters collaboration in children with ASD [59,78].

### Limitation and Future Works

Our findings should be interpreted in light of several limitations, which in turn inform a clear agenda for future research.

A key limitation of this study, reflecting a common challenge in ASD research [4], is the modest sample size (n=15 vs a planned n=25). This sample size was further constrained by our strategic decision to recruit from a single therapeutic center to ensure observational consistency. This approach was chosen to ensure that all sessions were conducted by the same group of participating therapists, who were deeply familiar with the children’s behavioral profiles, thereby ensuring observational consistency. While this strategy enhances the consistency of our observations, it could limit the generalizability of the findings, particularly given the cohort’s heterogeneity in chronological age (CA), functional age (FA), comorbidities, and ASD severity. In addition, only 8 of the 15 participants were retained for the ML analysis. Although the exclusions were based on data-quality and annotation considerations, some excluded sessions involved therapist assistance or interruptions. The retained analytic subset may therefore overrepresent children who could participate more independently and continuously, introducing potential selection bias. Consequently, the reported model performance may not represent the full study cohort or children with greater support needs. The study’s short-term nature is another limitation. Feedback from both caregivers and therapists highlighted the need for a longitudinal study to understand the long-term effects of using InteractiSense [81]. Prolonged exposure may be necessary for children to fully adapt to the novel device, potentially revealing usability benefits not apparent in a short-term evaluation and providing a more equitable comparison with highly familiar devices like the Xbox controller. However, this study did not examine longitudinal changes in collaborative task engagement, include standardized social outcome measures, or evaluate improvements in social skills. Accordingly, the current findings should be interpreted as evidence of the technical feasibility and short-term usability of InteractiSense rather than evidence of therapeutic effectiveness, intervention-related benefits, or improvements in collaborative task engagement or social skills.

Another limitation concerns the fixed physical dimensions of the prototype. Although the 18 cm measurement represents the outer diameter of the spherical enclosure rather than the required grip span, P9—the youngest participant and the participant with the shortest hand length, experienced some difficulty manipulating the device, suggesting that its overall form factor may have been relatively large for this child. The remaining participants were able to complete the assigned interactions using the device, but the small and heterogeneous sample did not permit a systematic assessment of usability differences by age or hand length. Future iterations should therefore evaluate smaller enclosure variants or adjustable gripping components to accommodate a broader range of users.

While securing large participant cohorts is a recognized constraint in special needs research, expanding the sample to establish the generalizability of these findings is essential. Future work should therefore address this limitation by validating our findings with a larger and more varied cohort over a longer period. Such a rich, longitudinal dataset would be crucial for advancing the engagement prediction model beyond its current, preliminary stage. It would enable not only a more definitive statistical analysis of user preference trends but also the systematic exploration of state-of-the-art (SOTA) architectures, feature engineering techniques, and parameters such as time-window size [82]. Furthermore, our poststudy interviews provided a clear, caregiver-driven roadmap for future iterations. A recurring theme was the need to enhance the device’s interactivity, for instance by adding light-up button cues or expanding its function into a responsive [6,9], playful companion that can generate its own sounds or haptic feedback [8]. On the gameplay side, caregivers advocated for a diverse and customizable library of SG content that can be tailored to the unique interests and needs of each child on the spectrum, such as incorporating preferred topics like numbers and puzzles or adjusting difficulty based on the child’s state [83]. InteractiSense is intended for researchers and practitioners in therapeutic and special education settings. It is not designed as a consumer product or a validated therapeutic intervention; rather, it serves as a proof-of-concept for examining the feasibility of multimodal sensing and data acquisition under controlled conditions, while also providing a preliminary investigation of how children with ASD engage with and respond to a novel multimodal device in terms of usability and interaction workload [84]. This work provides a methodological basis for future empirical studies examining the potential application of InteractiSense in therapeutic or adaptive contexts and its potential contribution to supporting collaborative task engagement.

### Conclusions

In this study, we proposed and evaluated InteractiSense, a tangible prototype designed to support interaction while enabling collaborative task engagement estimation through embedded sensing modalities, together with a collaborative and social SG intended to facilitate peer interaction among children with ASD. In a study involving 15 children, InteractiSense showed usability and workload levels comparable to those of a conventional Xbox controller, along with a tendency toward higher preference for the prototype. As the Xbox controller served as a familiar baseline shaped by longer-term use, whereas InteractiSense was evaluated after short-term exposure, this pattern provides initial support for feasibility at the interaction level. Signal quality varied across the embedded sensing modalities: ST yielded the strongest signals, HR and GSR were analyzable but susceptible to artifacts, audio quality was low, and pressure data were too sparse and discontinuous for analysis. Separately, a preliminary XGBoost model achieved 83% balanced accuracy in binary engagement classification, exceeding the Random Guess, Majority, and Logistic Regression baselines. However, these findings should be interpreted within the limits of the current sample, and the ML results in particular remain preliminary. Larger-scale and more longitudinal validation, together with expanded learning and sampling procedures, will be needed to clarify the robustness and generalizability of the observed performance.

## Supplementary material

10.2196/86264 Multimedia Appendix 1 Overview of the InteractiSense system and demonstration of treasure hunters gameplay.

10.2196/86264 Multimedia Appendix 2 Detailed descriptions of each data collected through InteractiSense.

10.2196/86264 Multimedia Appendix 3 Semistructured postexperiment interview questions administered to parents or caregivers.

10.2196/86264 Multimedia Appendix 4 Extreme gradient boosting hyperparameter search space and best selection.

## Abbreviations

ADHD: attention-deficit/hyperactivity disorder
ASD: autism spectrum disorder
CA: chronological age
ELAN: EUDICO Linguistic Annotator
FA: functional age
GIST: Gwangju Institute of Science and Technology
GSR: galvanic skin response
HR: heart rate
ID: intellectual disability
IRB: Institutional Review Board
ML: machine learning
NASA-TLX: NASA Task Load Index
OR: odds ratio
SG: serious game
SNR: signal-to-noise ratio
SOTA: state-of-the-art
ST: skin temperature
SUS: System Usability Scale
XGBoost: extreme gradient boosting
