# Supplementary material for: A Tangible Multimodal Sensing Interface for Collaborative Task Engagement in Children With Autism Spectrum Disorder: Cross-Sectional Mixed Methods Feasibility Study
Source: JMIR Serious Games. 2026 Oct 15;14:e86264. doi: 10.2196/86264 (PMC13671684; doi:10.2196/86264)
Supplement: Supplementary file 1 [file games-v14-e86264-s002.docx]

## Multimedia Appendix 2

The sensor configuration embedded into InteractiSense is described.

Detailed descriptions of each data collected through InteractiSense.

| Data Type | Data Sampling Rate | Data Range | Data Unit |
| --- | --- | --- | --- |
| Engagement | 60 Hz | 0/1 | Integer |
| GSR | 4 Hz | - | 𝜇S (Siemens) |
| HR | 30 Hz | - | bpm |
| ST | 4 Hz | - | ℃ |
| ACC (X, Y, Z) | 32 Hz | - | g ≈ 9.81𝑚/𝑠^2^ |
| Yaw, pitch, roll | 32 Hz | - | g rad |
| Gyro (X, Y, Z) | 32 Hz | - | g rad/s |
| Pressure | 30 Hz | 0~1 | Omega |
| Button | Every Input | 0/1 | Integer |
| Audio Amplitude | 30 Hz | - | dB |
