# Supplementary material for: A Tangible Multimodal Sensing Interface for Collaborative Task Engagement in Children With Autism Spectrum Disorder: Cross-Sectional Mixed Methods Feasibility Study
Source: JMIR Serious Games. 2026 Oct 15;14:e86264. doi: 10.2196/86264 (PMC13671684; doi:10.2196/86264)
Supplement: Supplementary file 2 [file games-v14-e86264-s003.docx]

## Multimedia Appendix 3

The semi-structured interview guide used to collect parents’ or caregivers’ observations and feedback after completion of all assigned experimental conditions is presented.

Semi-structured post-experiment interview questions administered to parents or caregivers.

| No. | Interview Question |
| --- | --- |
| Q1. | After using both InteractiSense and the Xbox controller to play Treasure Hunters, which device, if either, did the child appear to prefer? You may select InteractiSense, the Xbox controller, no observable difference, or difficult to determine. What behaviors informed your judgment? |
| Q2. | How did the child’s apparent enjoyment of the game differ, if at all, between the InteractiSense and Xbox controller conditions? What did you observe? |
| Q3. | How did the child’s ability to perform the game tasks differ, if at all, between the two device conditions? Please describe any differences you observed, including the child’s need for assistance or time to become familiar with each device. |
| Q4. | Which aspects of InteractiSense, if any, appeared to make interaction easier or more difficult for the child? Please describe both helpful and challenging aspects. |
| Q5. | During gameplay, what behaviors, if any, did you observe regarding the child’s participation in the shared task with the peer? Did these behaviors appear to differ between the two device conditions? Please provide specific examples where possible. |
| Q6. | How would you view the potential use of InteractiSense with compatible educational content or serious games at home? What benefits, concerns, or practical conditions would influence your willingness to use it? |
| Q7. | What changes, if any, would make InteractiSense or the collaborative game more suitable for the child? Where possible, please distinguish between changes to the device and changes to the game or content. |
