# Supplementary material for: A Tangible Multimodal Sensing Interface for Collaborative Task Engagement in Children With Autism Spectrum Disorder: Cross-Sectional Mixed Methods Feasibility Study
Source: JMIR Serious Games. 2026 Oct 15;14:e86264. doi: 10.2196/86264 (PMC13671684; doi:10.2196/86264)
Supplement: Supplementary file 3 [file games-v14-e86264-s004.docx]

## Multimedia Appendix 4

The XGBoost hyperparameter search space and best selections are summarized.

XGBoost hyperparameter search space and best selection. Best values are shown in **bold** and marked with *. ^a^

| Paramter | Search space | Best |
| --- | --- | --- |
| Learning rate (eta) | {0.03} (fixed) | **0.03*** |
| Max depth | {3, 4, 5} | **3*** |
| Min child weight | {1, 3, 5} | **1*** |
| Subsample | {0.6, 0.8, 1.0} | **0.8*** |
| Colsample by tree | {0.6, 0.8, 1.0} | **1.0*** |
| Gamma | {0, 1, 5} | **5*** |
| Regularization 𝛼 | {0, 0.5, 1} | **0.5*** |
| Regularization 𝜆 | {1, 5, 10} | **1*** |
| Scale_pos_weight | Computed from data | **0.0247*** |
| Num boosting rounds | Early stopping (≤2000) | **59*** |
| Threshold 𝜏 | Grid {0.05,0.5} | **0.3*** |

^a^*Notes*. Best configuration achieved Balanced Accuracy =0.81 on the validation set. Class imbalance was handled via *scale_pos_weight* and custom sample weights (minority and near-threshold emphasis). Objective was *binary:logistic*, eval metric was *logloss*, and tree method was *hist*.
